# True vignettes: interesting, illustrative examples of behavioral abnormalities in people with Parkinson's disease

**DOI:** 10.1055/s-0046-1822631

**Published:** 2026-05-18

**Authors:** Joseph H. Friedman, Umer Akbar, Blake Beehler, Kelvin L. Chou, Leslie J. Cloud, Andres Deik, Rebecca Gilbert, Stephen N. Gomperts, John L. Goudreau, Steven A. Gunzler, Karlo Lizarraga, Irene Malaty, Carine W. Maurer, Janis Miyasaki, Nisa Mohammed, John C. Morgan, Renato P. Munhoz, Gian Pal, Lisa Shulman, David Simon, Gregory Pontone

**Affiliations:** 1 Warren Alpert Medical School of Brown University, Butler Hospital, Department of Neurology, Providence RI, USA.; 2 Hackensack Meridian School of Medicine, Department of Neurology, Hackensack NJ, USA.; 3 University of Colorado, Anschutz Campus Medical School, Department of Neurology, Aurora CO, USA.; 4 University of Michigan, Department of Neurology, Ann Arbor MI, USA.; 5 Virginia Commonwealth University, VCU Health, Department of Neurology, Henrico VA, USA.; 6 University of Pennsylvania, Department of Neurology, Philadelphia PA, USA.; 7 American Parkinson Disease Association, Staten Island USA.; 8 Bellevue Hospital Center, New York NY, USA.; 9 Massachusetts General Hospital, Harvard Medical School, Department of Neurology, Boston MA, USA.; 10 Michigan State University, College of Osteopathic Medicine, Department of Neurology, East Lansing MI, USA.; 11 Michigan State University, East Lansing MI, USA.; 12 Case Western Reserve University, School of Medicine, Department of Neurology, Cleveland OH, USA.; 13 University of Rochester School of Medicine, Department of Neurology Rochester NY, USA.; 14 University of Florida, Fixel Institute for Neurological Disease, Department of Neurology, Gainesville FL, USA.; 15 Stony Brook University, Renaissance School of Medicine, Department of Neurology, Stony Brook NY, USA.; 16 University of Alberta, Department of Neurology, Edmonton AB, Canada.; 17 Rutgers-Robert Wood Johnson Medical School, Department of Neurology, New Brunswick NJ, USA.; 18 Medical College of Georgia, Department of Neurology, Augusta GA, USA.; 19 University of Toronto, Department of Neurology, Toronto ON, Canada.; 20 University of Maryland School of Medicine, Department of Neurology, Baltimore MD, USA.; 21 Beth Israel Deaconess Medical Center, Harvard Medical School, Department of Neurology, Boston MA, USA.

**Keywords:** Parkinson Disease, Hallucinations, Delusions, Compulsive Behavior, Lewy Body Disease

## Abstract

**Background:**

Parkinson's disease (PD) and dementia with Lewy bodies (DLB) are neurobehavioral disorders in which behavioral abnormalities are common but often not recognized.

**Objective:**

To bring to life the actual descriptions rendered by our patients of their unusual experiences.

**Methods:**

Members of the Parkinson Study Group were asked to provide illustrative examples of particularly memorable abnormal behaviors that their own PD patients or family members reported.

**Results:**

These recollections were deidentified and sorted into categories. These disorders include the classically described syndromes of Capgras, Cotard, Diogenes, Ekbom, Othello as well as overlap parasomnias, and impulse control disorders.

**Conclusion:**

Patients with PD often have had unusual experiences that they may not share without inquiries by their healthcare providers, fearful of how they will be perceived. Sensitive inquiries are required to reveal these problems. We believe that reading about real experiences will help sensitize the reader.

## INTRODUCTION

Parkinson's disease (PD) is classified as a movement disorder but almost always causes behavioral changes in the later stages. 1 These may be intrinsic to the disease, reactive to the pathology, or iatrogenic. It is uncommon for physicians other than neurologists to see a large number of PD patients, and few neurologists see sufficient cases to experience the full panoply of psychiatric complications that may occur.

As a result of the increasing numbers of movement disorders, neurologists have included behavioral aspects of PD in the portfolio of problems they treat so that fewer patients are referred to psychiatrists. This observation is supported by the percentage of papers on these topics authored primarily by movement disorders neurologists. The following reports illustrate problems that most non-movement disorders specialists rarely encounter, and are often hidden by patients. These were chosen because, while illustrative, they are interesting and memorable, in a way not captured in the general descriptions of these phenomena in texts and reviews.

Given the increasing number of elderly patients and, thus, people with PD, it is important for health care workers to understand this condition as a disorder that is more than an affliction of motor control. A similar collection was published in 2011. 2 We note that the distinction between PD with dementia and dementia with Lewy bodies (DLB) is often unclear, so we may have included some DLB cases, considering both as Lewy body dementias.

Hallucinations, mostly visual and without emotional content, eventually occur in the majority of treated patients, 1 3 4 5 while delusions tend to be paranoid in nature, and are, luckily, much less common. 1 3 4 5 Impulse control disorders occur in about 10 to 15% of those treated with dopamine agonists, 3 4 5 while a dopamine addictive syndrome (dopamine dysregulation syndrome, DDS) is very rare. 3 4 5 The rapid-eye movement (REM) sleep behavior disorder affects about 30% of men with PD, but much fewer women, often predates the onset of motor symptoms and is sometimes related to selective serotonin inhibiting (SSRI) drugs. 6 7 8 Other parasomnias, while documented in PD, remain poorly studied. 7 8

## METHODS

A series of clinical anecdotes was compiled by the lead author from neurologists and psychiatrists in the Parkinson Study Group to illustrate some of the more interesting/unusual from a wide range of behavioral changes. Patients' histories were not prospectively recorded for research purposes; rather, the clinical vignettes below were constructed from memory by the physicians who evaluated or treated these patients over the past 40 years.

Thus, clinical details, such as exact age, medications, duration and severity of disease, were not available for most cases. Additionally, names, ages, sometimes genders and other identifying details had to be changed in the descriptions below to meet the Institutional Review Board's (IRB) requirements. All of the quotes were recollections from one of the authors and were not exact.

A waiver of consent was approved by the Butler Hospital IRB for this article. The Brown University Regulatory and Advising Unit ruled that this work did not meet criteria for human research. After reviewing this manuscript, the board agreed that the reports described were sufficiently common, could be from anywhere in North America, and that more than one patient may have thought they were the subject. Thus, personal health information was not involved ( Tables 1 and 2 ).

**Table 1:** Syndromic names for neuropsychiatric syndromes

Cotard – delusion of being dead
Diogenes – self neglect without awareness
Ekbom – delusion of parasitosis
Hobbyism – compulsive pursuit of a hobby
ICD
Othello – delusional jealousy
Overlap – a parasomnia, that is similar to sleepwalking, with more complicated behaviors
Punding – compulsively taking things apart and putting them together

Abbreviation: ICD, impulse control disorder.

**Table 2:** Neuropsychiatric disorders by etiology

Disorder	Intrinsic	Iatrogenic
Psychotic symptoms	+	+
ICD		+
Parasomnias		+

Abbreviation: ICD, impulse control disorder.

## RESULTS

The vignettes are written as stories told by each author, hoping to show a more “real” and compelling side than if they had been presented in a uniform format.

### Hallucinations

Hallucinations are common, particularly in PD patients treated with medication. Minor or attenuated hallucinations, including fleeting visions in the peripheral field, feeling the presence of another person or animal, or visual illusions, may occur at any point in the illness, but are usually not perceived as an abnormality by the patients. 9 10 Additionally, they are not uncommon in neurologically normal individuals. 11 Formed, recurrent hallucinations in PD are generally visual, but less commonly, in descending order of frequency: auditory, tactile, olfactory, or gustatory. 12 They are usually emotionally neutral, even when the faces are distorted, or the figures are black and white. They are more common at night. Patients may have hallucinations, delusions, or both.

- A 68-year-old man was convinced that the water in his home was contaminated by water from Lake Erie, which caused his body to emanate an odor that attracted “Erie worms”. On my first visit with him, he had carefully captured and stored one of these worms to show me, slowly unfolding a tissue paper that was, of course, empty to my eyes but not his. He described the length (2–4 inches), color (green and yellow), and texture (slimy).

The worms infested his food, which was why he had trouble eating. They infested his sheets and pillows, which was why he had trouble sleeping. His wife often found sheets, pillows, and couch cushions thrown outside in the snow.

He made several calls to the county health department and water quality department to investigate his home and test his water, as well as having several visits from pest exterminators. When out in public, to the horror of his wife, he would alert patrons at a restaurant that their food had worms.

Comment: he saw the worms, so they were hallucinations; the odor may have been either hallucination or delusion; the infestation may have been delusional or hallucinatory.

- A 75-year-old, cognitively intact woman with PD for at least 18 years reported that, “My home was filled with people. They were dressed in costumes as if for Mardi Gras. I saw a guy on stilts, wearing a white tuxedo. Two children would peek out from behind the couch to watch what I was doing. People were everywhere.”
- This patient had two types of hallucinations. The first was described as the room flipped upside down. When someone comes into the room, it looks as if they are walking on the ceiling. If they sit down, hold her hand, and comfort her by saying the room is not upside down, her perspective changes and the room rights itself. The second one involves her hands. They look gnarled, with long, curled nails coming out of her fingertips, like claws. She hides them under her blanket, but when she uncovers them, there are little dragon heads between the claws. If someone comes and holds her hand, this hallucination goes away.
- A 68-year-old man is home alone each day. Several times per week in the midafternoon he sees a “parade of small wooden puppets marching through the house”. He describes the parade in great detail, some puppets wave streamers and others play instruments, though he hears no sound. He is not disturbed or upset by the puppets and, in fact, says he enjoys watching the parade, despite his brother's assurance that it is not real. One day, while watching the parade, he became hungry and decided to make a sandwich. He had to step over the parade on his way to the kitchen and fell, suffering a severe injury.

Comment: benign, possibly enjoyable visions may lead to bad outcomes.

- A 70-year-old man without dementia reported feeling a tiny piece of paper or material in his mouth for the several months, preceding the diagnosis of PD, but neither he, his dentist, or the otolaryngologist found an explanation.
- A 84-year-old woman without dementia was mildly distressed by a “black hole” appearing in her room, as well as in other rooms in her nursing home. She is afraid that people or things may fall in.

### Delusions

Delusions may be seen in association with hallucinations, but not always. Unlike hallucinations which tend to be without emotional content, delusions tend to be paranoid; often spousal infidelity, people living in the home, impostors posing as family, food being poisoned. 12 Like hallucinations, these are usually related to PD medication.

- An elderly man with PD for 15 years, on levodopa and rasagiline, living with his wife, became convinced that his wife “Natale” had run away with a man who lived in the apartment next door. She had arranged for an identical imposter in her place so he would not suspect anything. However, he was onto her because “new Natale” was taller than “old Natale.” He did not trust “new Natale,” so he started to refuse food, water, and medications from her, fearing he was being poisoned.

Roughly 2 weeks after starting quetiapine, the doctor asked him if “old Natale” was back. He replied “No, but I'm okay with it because ‘new Natale’ is actually very kind to me, and the fact that she is taller is a good thing because we are more matched in height.” At his next follow-up, I asked if “old Natale” was back? He replied “About that... there never was a new Natale. I was just really confused for a while.”

- A middle-aged woman believes there is a lizard occupying part of her intracranial space. She describes it as a bright green/deep brown lizard, that she can feel moving. It also feeds on the food she consumes by reaching the back of her throat using its fore limb. Although she is not demented, she has no insight that this is a delusion.
- Another male patient experienced the sensation of having large cockroaches coming out of his eyes. This patient had this delusion actively during the appointment, in front of the attending physician, and would apologize for the “disgusting sight” I was witnessing by having him in front of me, infested with insects.
- A patient reported that he awoke one morning feeling like a tiny man in an extremely big bed and had difficulty getting out of bed.
- Betty disclosed that Max, her long-time husband with PD, had delusions of infidelity. During a group exercise program at a nursing home, Max motioned for Betty to lean over. Betty did so readily, anticipating words of affection or appreciation. “Everyone here knows you're a whore.” Max had taken to telling all the nursing staff that his wife was a sex worker. Incredibly, some staff appeared to believe this. Betty continued to hope her husband would say something kind, but unfortunately, he passed away without acknowledging the incredible dedication and love she gave him. Betty could only say, “I know I did my best. That will have to do.”
- A 60-year-old woman with early-onset PD became manic shortly after deep brain stimulation (DBS) and claimed to have been raped while in the hospital.

### Impulse control disorders

The term impulse control disorders (ICD) is used to describe a wide spectrum of compulsive disorders associated with dopamine agonist medications, seen primarily in PD patients but also in other disorders in which these drugs are used, including restless legs syndrome and prolactinoma. While gambling and hypersexuality are the most common, several unique compulsions have been described. These are often not reported or minimized, so information from patients' friends and family should be sought.

- The patient was described as a kind, respectful, and wholesome man. When he developed an impulse control disorder, even his ICD was wholesome. He began frequenting a website called “cuddle comfort” where you can hire professional cuddlers to provide nonsexual hugs and cuddles for an hourly rate.
- I asked a woman on a dopamine agonist about a behavior she reported having developed on previous visits. She had developed an interest in vacuum cleaners that she found bizarre. “I needed a vacuum cleaner, went to the store and found them very interesting. When I got home, I decided that I needed a second one so I wouldn't have to carry it up and down the stairs. I thought my son also needed a vacuum cleaner for his home, so I got him one. I also got a small handheld one. I haven't bought any more, but whenever I go to the department store, I always check out the vacuum cleaners.”
- A 76-year-old retired man with PD reported that he kept busy by helping the widowed women in his apartment building with minor repairs. “What a great activity, for you to be helpful and socialize!” I encouraged. Shortly thereafter, I received an irate call from his son “Please stop encouraging him. He collects small appliances, disassembles them and can't put them back together, and we have to replace them! It's costing us an arm and a leg!”
- Another individual became hypersexual and very impulsive. I never saw him in an “off” state. His marriage was struggling. While away, I was forwarded an email by our clinic's nurse, with the note “Sorry to bother you on your holiday, but I think you should read this.” The email read “Dear Dr., I write to you from X Correctional Facility where I have been confined. This is a terrible misunderstanding. I was trying to talk to my wife and daughter who then locked themselves in the bathroom. I took the nearest tool at hand, a shovel and was trying to open the door. The police burst into my house and treated me like a criminal, handcuffing me without talking to me first.”
- This early-onset patient had bilateral subthalamic nucleus (STN) DBS. After 3 months, his wife and two children reported that he had become hypomanic (if not manic). Without consulting family members, he mapped out and planned a 33-hour walk to a local state park in the middle of the Northeastern winter. Fortunately, the patient's spouse became aware of this plan before he left the house, and the doctor was able to switch him back to his prior DBS settings, with resolution of mania.

### Dopamine dysregulation syndrome

As a rare subset of ICD, DDS is defined by levodopa addiction, with compulsive need for increasing doses of medication to feel “well” despite their recognition of the resulting negative consequences.

- A 64-year-old man developed DDS. He was arrested several times for waking his neighbors in the middle of the night, dressed in women's clothes, irate that he had not been invited to a party that, of course, was not happening. Later, in a nursing home where he was recovering from an illness, his bed was found to be full of hoarded carbidopa-levodopa (C-L) pills. He subsequently developed severe Guillain-Barre syndrome that rendered him quadriplegic. Despite being unable to move, he demanded as many C-L tablets as he could get, stating that they made him feel looser.
- A writer's ex-wife brought him to the emergency department, dyskinetic, sweating, with pressured speech. A call to his pharmacy allowed an estimated calculation of 3,000 to 5,000 mg of levodopa daily. Without my knowledge, he had escalated his dose of medication. His former wife went to his apartment and found bottles of water 3 feet apart throughout the entire living room, with a small pile of levodopa tablets next to the bottles. He would walk to a bottle, feel that he might freeze, and take 2 to 3 tablets. His last book was a disjointed collection of free-floating associations. A friend who visited thought the book displayed “genius”. During his hospitalization, he expressed grandiose ideation: being able to fly; or to cure himself of PD through mental fortitude, large dose of levodopa notwithstanding. Over 1-month hospitalization was required to taper his medication but was never in the “off”-state, even at discharge. Many years later, he remained hypomanic and dyskinetic, with periodic flares of psychosis.

Comment: the psychotic symptoms that developed, delusions of grandeur and special powers, are extremely atypical in PD.

### Parasomnias

Parasomnias in PD include REM Sleep Behavior Disorder (RBD), confusional arousals, and sleepwalking. Parasomnias other than RBD have attracted little attention. Furthermore, REM intrusions, as well as hypnopompic and hypnogogic hallucinations, are not included as parasomnias. While sleepwalking is usually a childhood disorder, an adult onset has been described in people with PD, being frequently associated with RBD. A review on this syndrome, 7 called “overlap syndrome” noted that “detailed characteristics of the phenomenon were not studied.” There were six patients described, who mostly walked around their homes, but one disrobed and “jumped around like a frog.” Little has been published since 2013, with most PD–sleep reports focused on RBD. The vignettes that follow fall into what the review describes as an “overlap” syndrome rather than sleepwalking or confusional arousals. 7

- An 82-year-old man, with mild dementia, was living in an assisted living facility (ALF) apartment located on the ground floor. One late cold winter night, with snow on the ground, wearing pajamas and slippers, he left his room searching for the start line for a race he was supposed to run. He thought he was in France, and walked into the woods adjacent to his apartment, looking for the starting line. Eventually he found himself on a street where a car stopped, asking where he lived, which he knew. He was delivered back to the ALF. He was thought to have been gone for almost 2 hours, but this was a guess. He spent a week in the hospital due to frostbite in his feet.
- A 75-year-old man with dementia and mild visual hallucinations was brought home by the police during the night. He described initially being lost in his house, having difficulty finding the stairs to leave his basement. He then went outside in his underwear and woke up a neighbor, informing her that he had lost something, though he wasn't sure what. In the doctor's office he noted that he had no basement and no stairs.
- A 72-year-old man was found walking alone on a street two miles from his home by his son-in-law who was driving home from work at 2 am . The patient didn't know where he was or how he got there.

## DISCUSSION

These vignettes bring to life the often-fantastic nature of patients' experiences, which are often puzzling to patients and family, but still feel real. It is uncommon for PD patients to volunteer information about hallucinations, delusions, or compulsive behaviors, sometimes worried about how others would view their sanity, sometimes embarrassed by their thoughts or behaviors, or even unsure these were not dreams. Therefore, clinicians need to routinely inquire about the presence of these phenomena. They are relatively benign at their onset but may worsen and cause great harm if not caught early.

A video production by the Parkinson's Foundation features other patients, not described in this paper, discussing their experiences was published on YouTube ( https://youtu.be/Gz2db91Ub54?si=ouYrp2-WPL_YT7Xx ).

A supplementary file containing more cases is available ( **Supplementary Material S1** – https://www.arquivosdeneuropsiquiatria.org/wp-content/uploads/2026/02/ANP-2025.0455-Supplementary-Material.docx ).
